# Manipulations of Libet clock parameters affect intention timing awareness

**DOI:** 10.1038/s41598-022-23513-1

**Published:** 2022-11-24

**Authors:** Bianca E. Ivanof, D. B. Terhune, D. Coyle, J. W. Moore

**Affiliations:** 1 Department of Psychology, Goldsmiths, University of London, New Cross Road, London, SE14 6NW UK; 2 Department of Computer Science, University College Dublin, Dublin, Ireland

**Keywords:** Consciousness, Human behaviour

## Abstract

W judgments are a widely used intention timing awareness estimate. These judgments are typically obtained by using the classic Libet-style paradigm whereby participants are asked to estimate the time they become aware of their intention to act by using the location of a rotating object on a clock face. There is an inconsistency in the Libet clock parameters used in previous studies, and it is unclear whether this variability impacts W judgments and other outcome measures, with implications for the construct validity of this measure and the generalisability of results across studies. Here, we present a four-experiment study that systematically manipulated the Libet clock speed, number of clock markings, length of the clock hand and type of clock radius in order to examine whether these parameter manipulations affect intention timing awareness estimates. Our results demonstrate W judgments can be significantly influenced by the clock speed and number of clock markings. The meaning and implications of these results are discussed.

## Introduction

Libet, Gleason, Wright and Pearl^1^ introduced intention timing awareness estimates ("W judgments") in an attempt to study conscious intention and the control of voluntary acts. In their study, participants attended to a clock face stimulus on which there was a rotating spot instead of a conventional clock hand. The spot completed one revolution of the clock face every 2560 ms and participants had to use its location on the clock’s perimeter to make three types of chronometric judgments—W judgments (i.e., the time they became aware of their intention to make an action), M judgments (i.e., the time they made an action) or S judgments (i.e., the time they became aware of an exogenous stimulus touch their skin). Simultaneously, the investigators recorded EEG activity from the participants’ scalps. Their central finding was that participants’ W judgments were reliably preceded by a gradual build-up of electrical brain activity (the readiness potential—“RP”), which led them to conclude that the brain initiates actions before we become consciously aware of our intention to act.

Following the introduction by Libet et al.^1^, W judgments have been used as a method to gain insights into different features of intention, highlighting the cognitive processes involved (e.g., Refs.^2–4^) as well as the possible neural correlates of intention processing (e.g., Refs.^5,6^). However, despite the widespread use of this measure, the methodology underlying the collection of W judgments has been criticised. For example, the validity and reliability of W judgments have been questioned, with some arguing that they are inaccurate, invalid or unreliable (e.g., Refs.^7–15^).

Beyond these criticisms, there are also concerns with the clock stimulus itself. One potentially important issue relates to the parameters of the Libet clock^16^. In particular, it is unclear where the original parameter settings came from (i.e., 2560 ms speed per clock revolution; clock markings in steps of five—5, 10, 15, etc.—a diameter with a visual angle of 1.8°). This uncertainty invites the question as to whether these configurations affect the dependent variables the clock is designed to measure, such as W judgments themselves.

A number of studies have examined the impact of Libet clock stimulus parameters on event timing judgments (e.g., Refs.^17–21^). For example, Pockett and Miller^20^ simultaneously manipulated seven different factors of the clock and assessed their impact on participants’ M judgments*.* They found only one of these factors (i.e., whether participants reported the start or end of their movements) to moderately affect participants’ M judgments, concluding that Libet’s method is robust. In contrast, a study by Danquah et al.^21^ showed that Libet clock manipulations can have a significant influence on S judgments. They changed the Libet clock speed across conditions (1280 ms vs 2560 ms vs 5120 ms per clock revolution) and found that participants’ S judgments varied as a function of clock speed. In light of these findings, Danquah et al.^21^ cautioned researchers against using this method.

More recently, Ivanof et al.^16^ examined whether manipulations of Libet clock parameters affect temporal binding, an implicit measure of sense of agency that uses the Libet clock to obtain timing judgments of actions and their effects^22^. Across five different experiments, they manipulated the clock speed (1280 ms v. 2560 ms v. 5120 ms per clock revolution), number of clock markings (no markings, markings in steps of 30′, 15′, 5′ and 5′ + 1′) and length of the clock hand (8 mm, 10 mm, 13 mm). They found changes in the Libet clock speed to significantly affect binding, with tone binding increasing in line with an increase in speed. Interestingly, this is a finding that parallels that of Muth et al.^45^, who examined similar aspects of an auditory Libet clock. Across four different experiments, they tested the influence of interval length, interval filling, sequence predictability and sequence length on temporal binding. They found that shortening the letter sequences (which can be regarded as a counterpart of the fast rotation speed in Ivanof et al.^16^) increased tone binding. Unlike Ivanof et al.^16^ however, they also found an increase in tone binding driven by a decrease in interval length (as a counterpart of fewer clock markings). This suggests that clock speed and resolution have the largest impact on temporal binding, which partly fits Ivanof et al.’s^16^ conclusions.

Despite these various strands of research, to our knowledge, no research has been conducted on the influence of changes in Libet clock parameters on W judgments. This is something the present investigation sets out to do, hereby complementing the work of Ivanof et al.^16^ and going beyond the aforementioned studies (i.e., Refs.^20,21^). We deem this to be important because, as stated above, W judgments remain a widely used intention timing awareness measure, and therefore any concerns about the stimulus-dependent nature of W judgments needs to be addressed. This is particularly pertinent given inconsistencies in the Libet clock settings used across studies. For example, there are differences in clock radius (e.g., Refs.^23,24^), in rotating object length (e.g., Refs.^25,26^) and in the number of clock markings (e.g., Refs.^27,28^). Thus, if the results of our investigation show that Libet clock settings manipulations affect W judgments, this would highlight the need for consistency in the choosing of these parameters, so that W judgments can be depicted accurately and results across the intention timing awareness literature are replicable. If, however, our results are statistically non-significant, this would show how Libet clock parameters can be modified to suit diverse methodological needs, without this affecting W judgments.

A further potential benefit from this investigation relates to the ongoing debate about the temporal relationship between the RP onset and W time. There has been some interest in how confounding factors may influence this relationship. For example, it has been suggested that the delay between RP onset and W time could be the result of certain biases (i.e., the representational momentum effect^29^; the flash-lag effect^30^; the prior-entry effect^31^). Furthermore, it has been shown that this temporal relationship is altered by the requirement to monitor the clock itself^32^ and also by variations in the type of clock used^33^. Our study will contribute to this debate by showing which clock parameters, if any, cause participants to make more or less anticipatory W judgments (which would imply a change in the temporal relationship with RP onset). This, in turn, carries downstream implications for debates around the usage of the Libet clock to probe the conscious initiation of voluntary acts (i.e., free will).

To this end, we conducted a four-experiment study to examine whether, and to what extent, systematically manipulating the Libet clock speed, number of clock markings, length of the clock hand and type of clock radius impacts on W judgments.

## General methods and statistical analyses

### Participants

Due to uncertainty regarding the anticipated effect size for the expected effect, we opted a priori to include 40 participants in this study, which would give us 80% power (assuming alpha = 0.05; sphericity = 0.8; three and five levels) to detect effect sizes of ɳ_p_^2^ = 0.03 and ɳ_p_^2^ = 0.27 (ɳ_p_^2^ provides an estimate of the amount of variance in the outcome attributable to the independent variable), respectively, and above for the main effects of our primary independent variables of interest (clock speed, number of clock markings, clock hand length and clock radius).

However, the COVID-19 pandemic prevented us from finishing the data collection, resulting in a final sample size of 26 participants (*M*age = 20.61 years, *SD* = 4.00, age range 19–37, no males). Participants were recruited using the Goldsmiths Research Participation Scheme and were compensated with 7.5 course credits for approximately 1.5 h of experimental time. One participant was subsequently excluded (see below “General methods and statistical analyses”); the remaining 25 participants (*M*age = 19.84 years, *SD* = 5.68, age range 19–37, no males) were all right-handed, had normal or corrected-to-normal vision, with no self-reported psychiatric or neurological disorders or substance usage that might interfere with their cognitive performance. They provided written informed consent prior to participation, and the experiment was approved by the Department Ethics Committee at Goldsmiths, University of London and it was conducted in accordance with relevant guidelines and regulations.

### Materials

Intention awareness time was measured using a Libet clock task programmed in JAVA (version 6; ORACLE, 2011). The speed at which the clock hand rotated, the number of clock markings, the length of the clock hand and the radius of the clock face changed across experiments (see below “Methods” per each experiment). The clock hand always stopped rotating after 1000–2500 ms following the action (see below).

**Figure 1:**
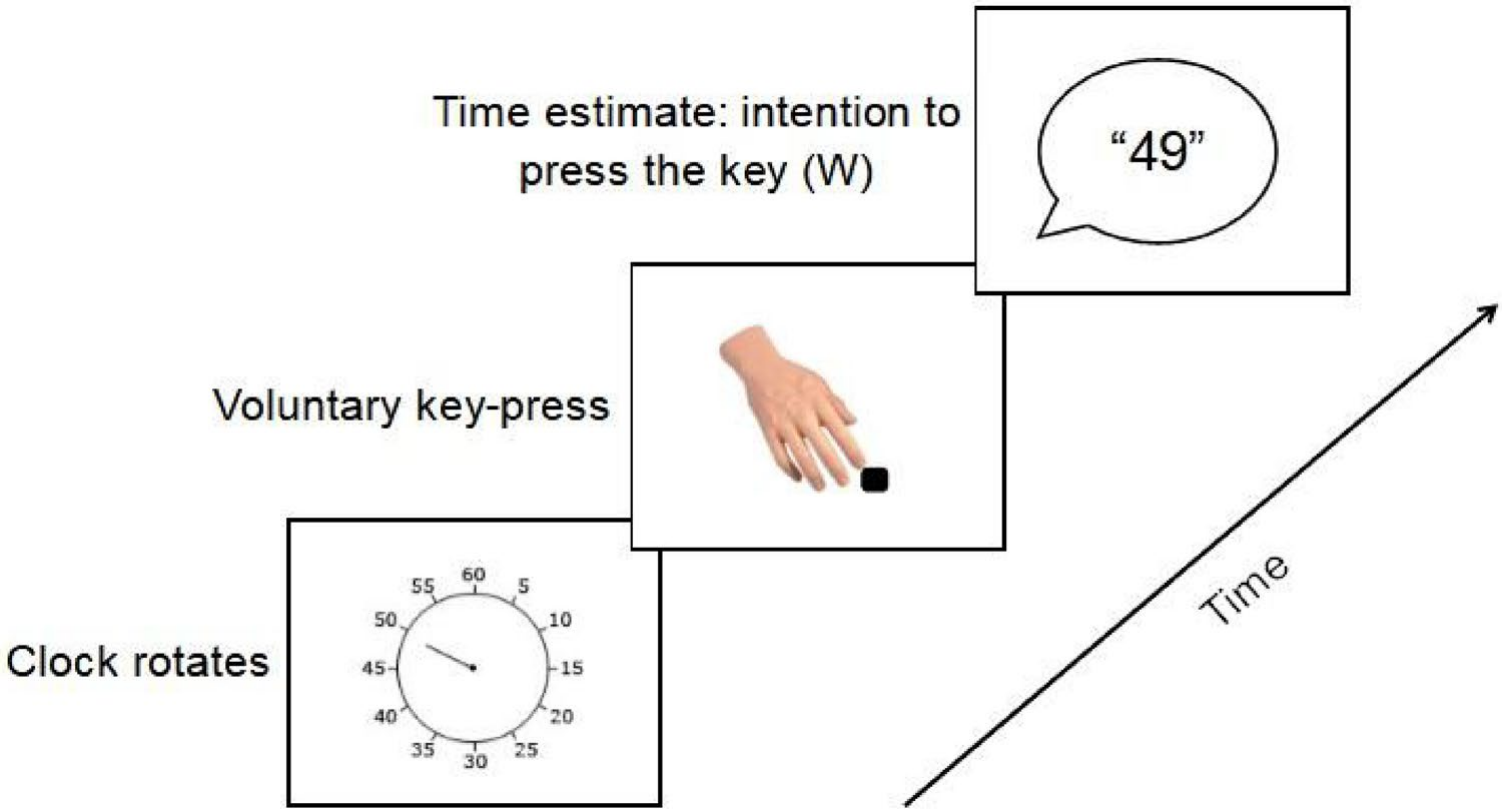
Standard trial structure across all experiments. Participants were required to press a key at their own pace and report the time they first became aware of their intention to press the key.

As can be seen in Fig. 1, participants were instructed to sit in front of a computer depicting a standard Libet clock and press a pre-specified key with their right index finger whenever they felt like it. They were asked to report the moment at which they first became aware of their urge to press the key. To make this judgment they were instructed to estimate where on the clock face the clock hand was pointing to when they had the urge to press the key. They verbally reported this number to the experimenter at the end of the trial, who entered it on their behalf.

### Procedure

All four experiments presented in this paper (i.e., clock speed, number of clock markings, clock hand length and clock radius) were run in a single session. These were counterbalanced across participants and the blocks present in each experiment (i.e., three clock speed, five clock markings, three clock hand length and three radius blocks) were randomised within participants. Each block contained 30 trials. Out of these 30 trials, the first three were always practice trials participants completed.

Participants sat at a distance of approximately 65 cm from the clock face across all experiments (visual angle: 1.8° with the exception of two Clock radius blocks—see below “Methods” section of “Experiment 4”), were informed of any change in clock parameters before completing a new experiment and block, were given opportunities to rest in-between experiments and were reminded to be as accurate as possible when reporting their intention time awareness estimates.

### Statistical analyses

The variable of interest here was W judgments, which correspond to the difference in time between participants’ estimated intention to move and the time of their actual key-press. This measure was computed on each trial and then averaged across trials, to give an average W judgment for each block per experiment per participant.

Individual trials containing W judgments outliers within participants were excluded (M ± 2.5 SDs; see below “Statistical analyses” sections of all experiments). Outliers at the group level were removed if a combination of factors indicated univariate or multivariate outliers (Ref.^34^; these included skewness and kurtosis values greater than approximately ± 2.000, significant results rendered by the Kolmogorov–Smirnov test, visual inspection of boxplots and histograms). These criteria resulted in the exclusion of one participant, who was excluded on the basis of boxplot inspection and extreme kurtosis values.

For all experiments, we used repeated-measures ANOVAs and Bonferroni-corrected post-hoc comparisons where appropriate. Whenever the data were non-normally distributed, we used Friedman’s ANOVA and Bonferroni-corrected Conover’s post-hoc tests. All analyses were supplemented by estimates of effect size (ɳ_p_^2^ and Cohen’s *d*) and by Bayes Factors with default priors. BFs represent a measure of the relative likelihood of one hypothesis relative to the other given the available data^35^. Following convention, we interpreted BFs greater than 3 as providing moderate (or greater) evidence for the alternative hypothesis, less than 0.33 as moderate evidence for the null hypothesis, and between 0.33 and 3 as insensitive evidence^36,37^. Finally, to probe the within-participants reliability of W judgments across all clock manipulations, we computed Spearman’s rho correlation coefficients among all mean W judgments per block per experiment, which we corrected for multiple comparisons using the FDR method^38^.

All data and statistical analyses were conducted using MATLAB (v. R2012a, MathWorks, Natick, MA), JASP (v. 0.16.2) and GPower (v. 3.1).

## Experiment 1: Clock speed manipulations and W judgments

Ivanof et al.^16^ demonstrated how an increase in clock speed can affect temporal binding by increasing tone binding. Moreover, Danquah et al.^21^ have showed that an increase in clock speed also appears to make S judgments significantly less anticipatory. On the basis of these results, we predicted that an increase in the Libet clock speed will make W judgments significantly less anticipatory. Using the same rationale and methodology as Ivanof et al.^16^, we asked participants to make W judgments by using a clock with different clock hand rotation speeds (1280 ms v. 2560 ms v. 5120 ms per clock revolution).

## Methods

### Materials and procedure

As part of this experiment, the clock measured 21 mm in diameter, featured a 9 mm hand, was marked at conventional intervals (5, 10, 15, etc.) and rotated at three different speeds across three separate blocks—1280 ms (fast), 2560 ms (the standard rotation speed used in the field) and 5120 ms (slow) per clock revolution. Participants spent approximately five minutes completing each block (15 min for the entire clock speed experiment).

### Statistical analyses

Due to the data being non-normally distributed, we conducted Friedman’s ANOVA with Clock speed (1280 ms, 2560 ms, 5120 ms) as the within-subject factor and mean W judgment as the dependent variable. We ran Bonferroni-corrected Conover’s post-hoc tests, which were supplemented by Cohen’s *d*s for estimates of effect size and by Bayes Factors with default priors. We removed 1.20%, 1.60% and 2.26% trials from the 1280 ms, 2560 ms and 5120 ms clock speed condition, respectively.

### Results and discussion

The analyses revealed a significant main effect of Clock speed on mean W judgments, χ^2^(2, 25) = 14.48, *p* < 0.001, *W* = 0.29, BF10 = 8174.13. Bonferroni-corrected Conover’s post-hoc tests revealed significant differences in mean W judgments between the 1280 ms v. 2560 ms, *p* = 0.03, *d* = -0.95, BF10 = 80.90, and the 1280 ms v. 5120 ms, *p* = 0.002, *d* = -1.05, BF10 = 86.39, yet a medium-sized trend for significance between the 2560 ms v. 5120 ms conditions, *p* = 0.98, *d* = 0.72, BF10 = 7.73 (see Fig. 3a). As predicted, this suggests that increasing the clock speed affects W judgments by making them significantly less anticipatory. This result echoes that of Danquah et al.^21^, who found that S judgments too become less anticipatory in line with an increase in speed, and alludes to the conclusions reached by Ivanof et al.^16^, who found that a faster clock speed increases tone binding.

Given that much has been made of differences in the timing of W judgments in relation to other events—notably, RPs in the brain—our findings highlight the need for caution in the interpretation of experiments involving the collection of W judgments, especially as this has a bearing on the link between conscious intention and the control of voluntary acts. Moreover, similarly to the conclusions reached by Ivanof et al.^16^ in the context of temporal binding, this result emphasises the need for consistency in this Libet clock parameter across the intention timing awareness literature. In the next experiment we assess the influence of another clock feature (clock markings) on W judgments.

## Experiment 2: Clock markings manipulations and W judgments

This experiment focusses on the potential effect of the number of clock markings on W judgments. As Ivanof et al.^16^ show, in the context of temporal binding research there is a degree of inconsistency in the number of clock markings presented on the Libet clock. This is also true of studies on intention timing awareness. For example, some investigators use a clock marked in steps of 5′ with additional radial lines displayed in steps of 2.5′ (e.g., Refs.^1,28^), others a clock marked in steps of 5′ only (e.g., Refs.^25,39^), or a clock marked more granularly, in steps of 1′ (e.g., Refs.^24,27^). Some authors modified the clock substantially and used a rectangular clock marked at 50 equally spaced positions, with four additional tic marks appearing in the corners of the rectangle (e.g., Refs.^32,40^).

In order to shed light on the putative effect of the number of clock markings on W judgments and its implications for the W judgments literature, we used the same types of clock markings as Ivanof et al.^16^ (no markings—here called “none”—clock marked in steps of 30′, 15′, 5′ and 5′ + 1′). Our predictions regarding the effect of these manipulations on W judgments were non-directional.

## Methods

### Materials and procedure

**Figure 2:**
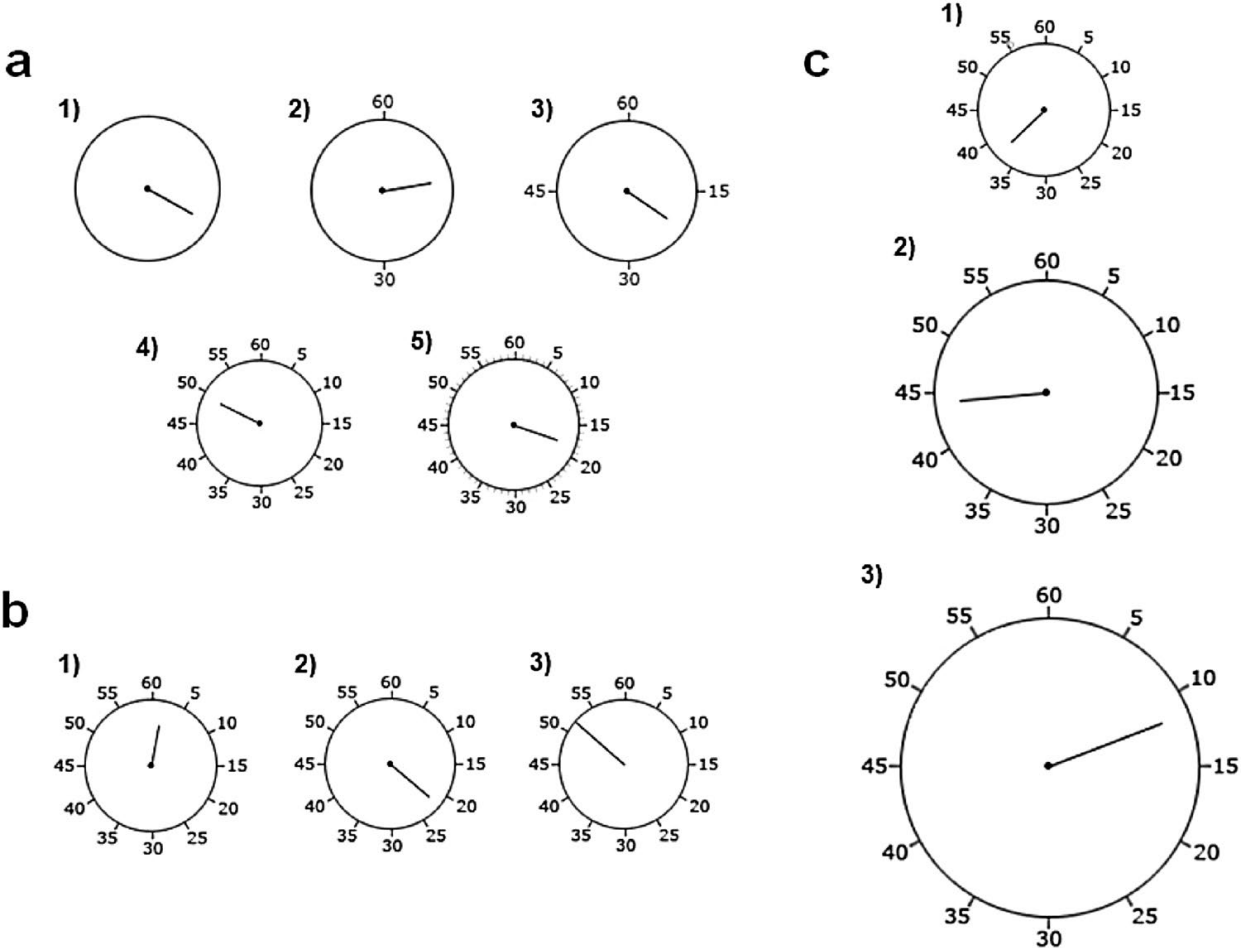
Three of the clock manipulations used across this four-experiment study. Panel a contains all the clock markings manipulations (1–none, 2–30′, 3–15′, 4–5′, 5–5′ + 1′), panel b all the clock hand length manipulations (1–8 mm, 2–10 mm, 3–13 mm) and panel c all the clock radius manipulations (1—small radius, 2—medium radius, 3—large radius).

For this experiment, the clock rotated at the standard speed of 2560 ms per revolution, featured a 9 mm hand, measured 21 mm in diameter and the clock face contained five types of clock markings across five separate blocks (none—the clock was not marked at all; 30′—the clock was marked in steps of 30′; 15′—the clock was marked in steps of 15′; 5′—the clock was marked in steps of 5′; 5′ + 1′—the clock was marked in steps of 5′ and in more granular steps of 1′ (thus containing 60 markings); see Fig. 2a—a_1_, a_2_, a_3_, a_4_, a_5_, respectively). Participants spent approximately five minutes per block (a total of 25 min for this experiment).

### Statistical analyses

We conducted a one-way repeated-measures ANOVA with Clock markings (five levels) as the within-subject factor and mean W judgment as the dependent variable, and Bonferroni-corrected pairwise comparisons with Cohen’s *d*s as estimates of effect size. We supplemented these analyses with Bayes Factors with default priors. This time, 1.73%, 1.60%, 2.13%, 3.06% and 1.60% of trials from the none, 30′, 15′, 5′ and 5′ + 1′ conditions, respectively, were removed.

### Results and discussion

**Figure 3:**
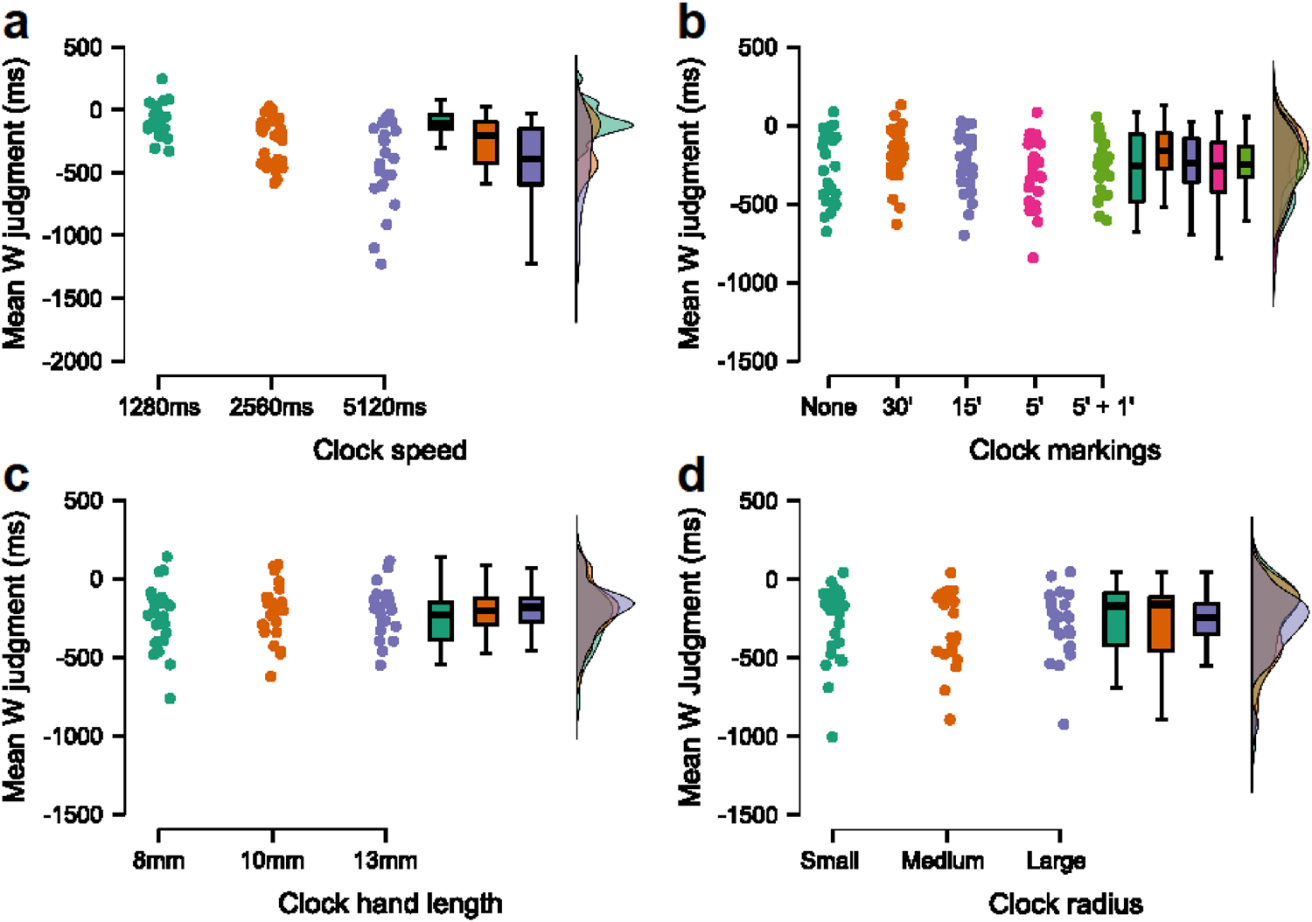
Results across all four experiments. Panels (**a**–**d**) contain the rain plots showing the results of the Clock speed, Clock markings, Clock hand length and Clock radius experiments, respectively.

The one-way ANOVA showed a significant main effect of Clock markings on W judgments, *F*(4, 96) = 4.43, *p* = 0.002, ɳ_p_^2^ = 0.156, BF10 = 12.88. Bonferroni-corrected pairwise comparisons revealed a significant difference in W judgments between the none and 30′ condition, *p* = 0.026, *d* = − 0.407, BF10 = 11.48, and the 30′ and 5′ condition, *p* = 0.001, *d* = 0.52, BF10 = 130.35. There was also a trend-level, medium-sized difference in W judgments between the 30′ and 5′ + 1′ condition, *p* = 0.051, *d* = 0.377, BF10 = 5.30. No other comparisons approached significance, all *p*s > 0.05, *d*s < 0.308, BF10s < 2.12 (see Fig. 3b).

These results suggest that the number of clock markings affects W judgments in such a way that, when the clock has a minimal amount of detail displayed (i.e., the 30′ condition), as opposed to no detail at all (i.e., the none condition) or a lot of detail (i.e., the 5′ and 5′ + 1′ conditions), these judgments are more anticipatory.

This finding does not seem to mirror that of Ivanof et al.^16^, who found no significant effect of manipulations of Libet clock markings on temporal binding. It, thus, might speak to how variations in this clock parameter can affect the comparability of results across the intention timing awareness literature. Moreover, given that this result implies that W judgments are more or less closer to the RP onset or to M time depending on the number of Libet clock markings used, this also brings to light a further factor that might cloud the interpretation of the relationship between W judgments and other variables (such as the RP). In the next experiment, we turn our attention to another Libet clock parameter—the clock hand length—and its possible influence on W judgments.

## Experiment 3: Clock hand length manipulations and W judgments

This experiment focusses on the influence the length of the clock hand might have on participants’ W judgments. Ivanof et al.^16^ investigated this issue in the context of temporal binding and found no significant effect of clock hand length on either action or tone binding. Nonetheless, this is another parameter that varies across the intention timing awareness literature as well (e.g., a clock hand measuring 12 mm^25^; 17.5 mm^39^ or equal to the radius of the clock^26^). Moreover, this parameter is widely under-reported, which further highlights the need to study the effect it might have on W judgments.

Similarly to Experiment 2, our predictions regarding the effect of these three different clock hand lengths on W judgments were non-directional.

## Methods

### Materials and procedure

For this experiment, the clock hand rotated at 2560 ms per revolution, the clock face was marked in steps of 5′, measured 21 mm in diameter and the clock hand length varied across three blocks (8 mm, 10 mm, 13 mm; see Fig. 2b—b_1_, b_2_ and b_3_, respectively). Participants spent five minutes per block (15 min in total).

### Statistical analyses

We analysed the data using a one-way repeated-measures ANOVA with Clock hand length (8 mm, 10 mm, 13 mm) as the within-subject factor and mean W judgment as the dependent variable. We supplemented these analyses with Bayes Factors with default priors. 1.33%, 1.60% and 1.60% of trials were removed from the 8 mm, 10 mm and 13 mm condition, respectively.

### Results and discussion

The one-way ANOVA revealed no significant main effect of Clock hand length on W judgments, *F*(2, 48) = 1.45, *p* = 0.25, ɳ_p_^2^ = 0.06, BF10 = 0.33 (see Fig. 3c). This suggests this particular Libet clock parameter does not significantly affect intention time awareness estimates.

This implies that variations in clock hand length are unlikely to represent a confound for the comparability of results across the intention timing awareness literature, which is a result that echoes that of Ivanof et al.^16^ in the context of temporal binding. This also implies that manipulations of this clock parameter are unlikely to significantly inform debates revolving around conscious intention and the control of voluntary acts in Libet-style experiments.

## Experiment 4: Clock radius manipulations and W judgments

Here, the main point of focus was the clock radius. This is a clock feature whose impact on temporal binding was not considered by Ivanof et al.^16^. However, intention timing awareness investigators vary in their preference for clock radius too, which makes this issue a topic worth investigating empirically. For example, some researchers have used larger Libet clocks (e.g., 74.3 mm diameter^23^; 90 mm diameter^27^; 101.6 mm diameter, visual angle: approximately 9.6°^41^) while others have used smaller clock faces (e.g., 13 mm diameter^42^; 35 mm diameter, visual angle: 4°^39^; 40 mm diameter^24^).

As such, this experiment’s aim is to look into how different types of clock radius affect W judgments and, thus, to shed light on whether inconsistencies in this Libet clock feature might represent a confound for the intention timing awareness literature. Our predictions regarding the effect of clock radius on W judgments were non-directional.

## Methods

### Materials and procedure

The clock rotated at the standard speed of 2560 ms per revolution and was marked in steps of 5′. We used three different clock radii across three blocks. The clock hand length increased in accordance with an increase in radius so as to preserve the clock face-to-clock hand ratio (small radius: 10.5 mm, clock hand length: 9 mm, visual angle: approximately 1.8°; medium radius: 17.5 mm, clock hand length: 13 mm, visual angle: approximately 3.1°; large radius: 23.5 mm, clock hand length: 19 mm, visual angle: approximately 4.1°; see Fig. 2c—c_1_, c_2_, c_3_, respectively). As before, participants spent approximately five minutes completing each block (15 min in total).

### Statistical analyses

We ran, as before, a one-way ANOVA with Clock radius (small, medium, large) as the within-subject factor and mean W judgment as the dependent variable, and calculated Bayes Factors with default priors. We excluded 1.86%, 2% and 2.53% of trials from the small, medium and large radius condition, respectively.

### Results and discussion

The one-way ANOVA revealed no significant main effect of Clock radius on mean W judgments, *F*(2, 48) = 0.21, *p* = 0.81, ɳ_p_^2^ = 0.01, BF10 = 0.13 (see Fig. 3d). This suggests that the clock radius did not significantly impact participants’ intention timing awareness. This might imply that this particular clock feature can be manipulated without the risk of influencing W judgments. This also implies that manipulations of the Libet clock radius are unlikely to inform the interpretation of the link between conscious intention and the control of voluntary acts in Libet-style experiments.

## Within-participants reliability of W judgments

Finally, due to this study using the same participants across all experiments and given the concerns with the reliability of W judgments certain investigators have recorded to date (see “Introduction”), we took advantage of the opportunity of testing the intra-participants reliability of W judgments. As such, we computed Spearman’s rho correlation coefficients between all mean W judgments per block per experiment, correcting the associated p-values using the FDR method^38^.

### Results and discussion

**Figure 4:**
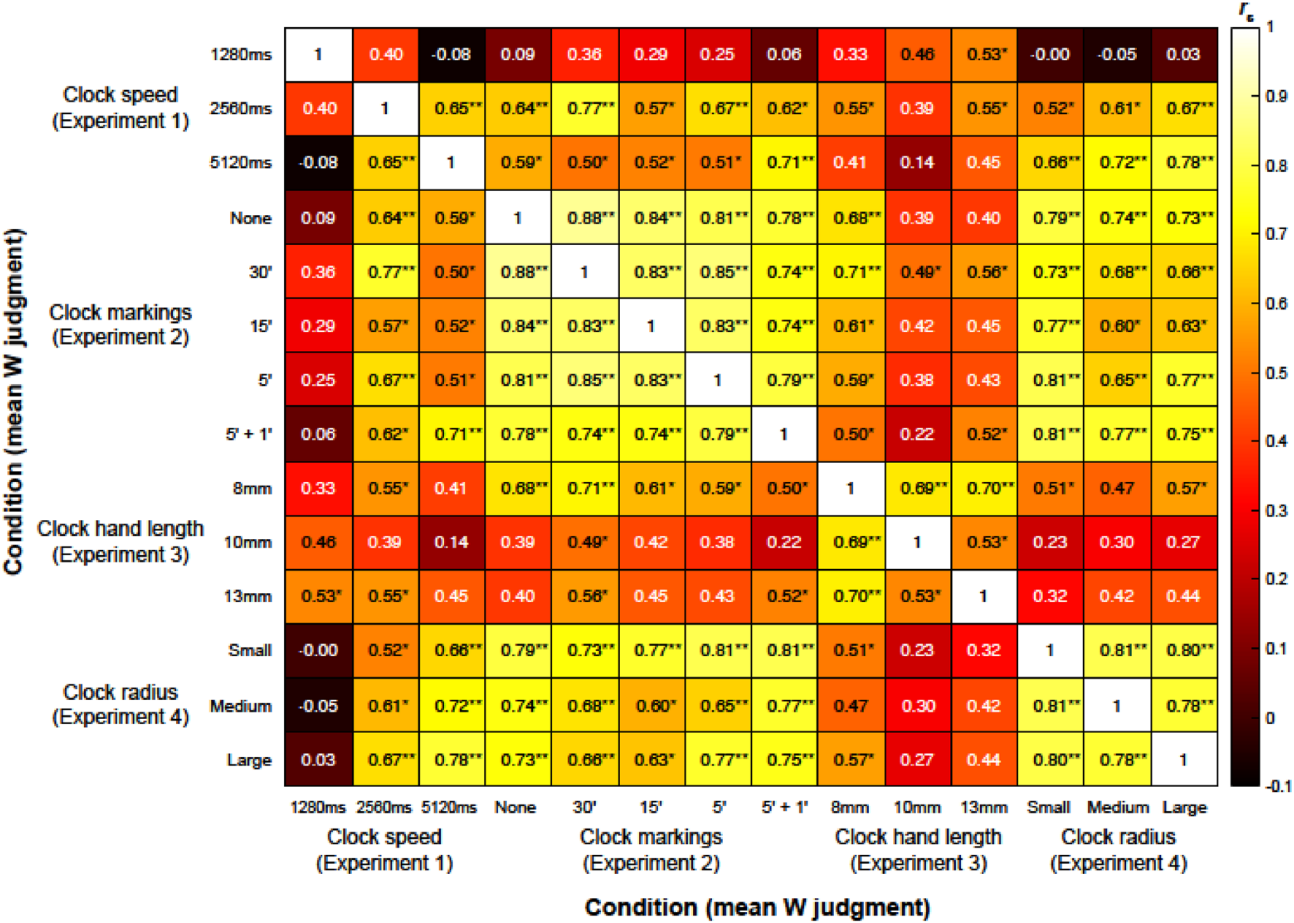
Heatmap containing the Spearman’s rho correlation coefficients between all mean W judgments per block per experiment. * and ** represent p < 0.013 (FDR) and p < 0.001, respectively.

As can be seen in Fig. 4, W judgments seem to be reliable across the majority of blocks and experiments. However, as the heatmap above shows, Spearman’s rho coefficients correlating W judgments in the 1280 ms clock speed block with all the other blocks are largely non-significant and small-sized. This suggests that such a high clock rotation speed does not permit an equally reliable reporting of W time (as the other blocks do). Also, a more subtle effect is that of W judgments in the 8 mm clock hand length block, which seem to be more reliable than those in the 10 mm or 13 mm blocks (as the former correlate with other blocks more strongly). As such, we advise researchers to avoid the 1280 ms rotation speed clock setting and consider using clock hands that measure 8 mm.

## General discussion

This four-experiment study investigated the effect the Libet clock speed, number of clock markings, length of the clock hand and type of clock radius have on intention timing awareness estimates (W judgments). As part of the standard Libet clock experimental setup, participants were asked to report the time they became aware of their urge to press a key. Our results suggest that W judgments can be significantly affected by the rotation speed of the clock, this result alluding to similar effects of clock speed on tone binding^16^ and on S judgments^21^. Moreover, W judgments can also be affected by the number of clock markings, which does not seem to be the case with temporal binding as seen in Ivanof et al.^16^. Finally, correlation coefficients computed between all mean W judgments per block per experiment hinted towards the fact that W judgments made in the 1280 ms clock speed block are not reliable and that W judgments made in the 8 mm clock hand length block are more reliable than those made in the 10 mm or 13 mm blocks. Below we elaborate on the implications of these results.

### Practical implications

Our findings imply that the inconsistency in and under-reporting of Libet clock parameters used can affect the comparability of results across the intention timing awareness literature and, consequently, mar replication attempts. Similarly to Ivanof et al.’s^16^ conclusions, this is less problematic in the case of the Libet clock speed, as this feature tends to remain constant (the standard speed used in most studies is ~ 2560 ms per clock revolution). Nevertheless, this can indeed pose problems when it comes to the number of clock markings, as this parameter varies.

At the opposite end of the spectrum, our results suggest that the clock radius can indeed be manipulated without the risk of it influencing W judgments. For instance, the clock could be larger so as to minimise the perceptual burden of the task when testing certain populations in whom this aspect of the task might be a problem, such as in specific psychiatric or neurological patients or certain age groups (e.g., older adults or younger children). It may also be the case that this alteration of the clock stimulus would be advantageous when testing typical populations, if it reduces perceptual/cognitive fatigue (which is a concern with these tasks, as trial numbers can often be quite high).

### Theoretical implications

Our findings point to the stimulus-dependent nature of W judgments—changes in the Libet clock speed and number of clock markings can significantly alter the time at which people perceive their intention to act. This is noteworthy given that the temporal relationship between W judgments and the readiness potential (RP) has attracted great interest. Libet et al.^1^ showed that there is a delay between the onset of the readiness potential and that of W judgments. Some have taken this to pose a challenge for the notion of free will, as it implies that our conscious intentions do not cause our actions (e.g., Refs.^43,44^). Importantly, our findings suggest that the delay between RP and W judgments can be influenced by the setting of the stimulus used to measure W itself. Whether or not this effect can fully explain the delay is currently unclear, but it should certainly encourage a degree of caution in the interpretation of this delay and any theoretical implications drawn from it.

In this way, our findings add to the range of phenomena that may lengthen the duration between the RP and W judgments (e.g., the representational momentum effect^29^; the flash-lag effect^30^; the prior-entry effect^31^). The current results also add to the work of other investigators that highlight how the act of monitoring the clock affects W judgments^32^ or how the type of clock used affects the lag between M and W judgments^33^.

### Limitations and future directions

As noted above in the “General methods and statistical analyses” section, testing for these experiments was halted by the COVID-19 outbreak. As such, the sample size is only sufficiently powered to detect effects in the range of ɳ_p_^2^ = 0.03 and ɳ_p_^2^ = 0.27. This is relevant given that there were two weak trends in the data that did not reach significance (i.e., 2560 ms v. 5120 ms clock speed condition, 30′ v. 5′ + 1′ clock markings condition). It is plausible that these effects would have achieved significance with a larger sample size. As such, we advise future research to replicate our findings in the context of a bigger sample size.

It is also worth emphasising that we did not collect EEG data and were therefore not able to measure preparatory motor signals (i.e., RPs). Although not essential for the purposes of our experiments and the manipulations that we evaluated, such data would have afforded us greater certainty with regard to how changes in W relate to RPs. Without these data, our suggestions are merely speculative at this stage, and they would constitute something for future research to address.

Finally, we deem it right to highlight that, at times, our choice of clock settings could have been better motivated. For instance, we used 8 mm, 10 mm and 13 mm in the Clock hand length experiment, but 9 mm in the remaining experiments. Despite this issue, our manipulations still allowed us to address the fundamental question of whether or not this parameter influences W.

### Conclusions

Here we have systematically examined the effect of Libet clock manipulations on intention timing awareness. We have shown that clock speed and the number of clock markings significantly influence W time, but that the clock radius and clock hand length do not. It is our hope that these findings will not only help inform the design of future experiments using the Libet clock to probe W time but will also contribute to the ongoing debate concerning the relationship between RP onset and W time.

## Data Availability

The datasets generated and analysed during the present studies are available on Bianca E. Ivanof’s OSF profile at osf.io/7z3xd, under the “Quick files” section, inside the “Ivanof et al. W judgments and Libet clock” folder.
